# On the Noise Effect of Fingerprinting-Based Positioning Error in Underwater Visible Light Networks †

**DOI:** 10.3390/s21165398

**Published:** 2021-08-10

**Authors:** Marwan Hammouda, Anna Maria Vegni, Valeria Loscrí

**Affiliations:** 1 Indenpendent Researcher, 38114 Brunswick, Germany; marwan.hammouda@gmail.com; 2 Department of Engineering, Roma Tre University, 00146 Rome, Italy; 3 INRIA Lille-Nord Europe, 59650 Lille, France; valeria.loscri@inria.fr

**Keywords:** visible light communications, underwater positioning, accuracy, noise error

## Abstract

This paper assesses the performance of a localization technique for underwater visible light networks. The proposed approach is based on a fingerprinting technique, collecting the channel impulse responses from different wireless optical signals in the visible range. A local database related to the power level distribution within a maritime environment is built and exploited to estimate user position, e.g., a diver moving in a given space for underwater fish monitoring. In this paper, we investigate on the noise effect on the localization accuracy in underwater scenarios and for different water turbidity coefficient and we demonstrate that the estimation error suffers on variable channel impulse responses. Different configuration parameters and environmental scenarios have been considered, showing that the LED transmitter deployment can be effective in the localization estimation. A comparison of the proposed localization approach to the traditional triangulation method has been finally carried out, showing the effectiveness of the fingerprinting-based solution for a lower number of LED transmitters.

## 1. Introduction

Great expectations are relied on future 6G networks for ensuring extremely high capacity and enabling new emerging applications [1]. Current technologies cannot meet these requirements and, above all, 6G networks are supposed to integrate air/space/underwater wireless systems by means of terrestrial networks. The overall coverage will be improved through 6G networks, by providing the high-data rate that traditional wireless networks cannot guarantee.

In more detail, 6G networks are supposed to support transmission rates from 100 to 1000 times higher than those for 5G [2]. In this regard, one of the communication technologies regarded with much interest is Visible Light Communication (VLC) with a working frequency range of [400,800] THz. Among the recent hot research topics, the underwater VLC (UVLC) can be observed as an enabler of the Internet of Underwater Things paradigm (IoUT) [3,4]. IoUT is a recent paradigm relying on the fundamental consideration that water covers more than two-thirds of Earth’s surface in the form of oceans. Several factors related to human beings are based on oceans, e.g., transportation, climate regulation, food supply chain and many others. At the moment, oceans represent a highly challenging environment and, as a matter of fact, most oceans remain unexplored.

IoUT can be considered as a revolutionary technology not only in the case of ocean exploration but for its biodiversity maintenance as well. Alternative communication technologies need to be considered in such a type of environment due to the harsh conditions in oceans and water. Among these technologies are magnetic induction, acoustic waves and optical waves, just to mention a few. These communication mediums present some advantages for data transmission but, at the same time, experience under performance degradation due to the various effects of harsh underwater environments [5]. Acoustic waves [6,7] are typically considered for their capability of reaching long distances with stable connectivity, however, acoustic links suffer low data rates over relatively long distances and, thus, strongly limit data transmission and infers longer delays. Typically, data sensed from underwater sensor networks are interpreted with reference to the sensor position, e.g., by tracking the movement of a target object and monitoring a certain area. In such scenarios, underwater localization is very challenging, since well-known Global Positioning System (GPS) technology cannot penetrate.

Generally, localization schemes are based on the knowledge of the position of some nodes, referred as anchor nodes or reference nodes [8]. In [9] a scheme named 3-DUL is proposed where three anchor nodes are used for generating the global position in a 3D space. The proposed 3-DUL approach is based on two phases where the first one is for nodes with unknown positions to find the distance to the neighboring anchor nodes and the second one is based on the exploitation of the pairwise distances and depth information in order to protrude the anchors in the horizontal plane and to generate a virtual geometric shape. Based on the shape, this node is capable to self-positioning by the means of a dynamic trilateration, thus, becoming an anchor node. This process is iterated in a dynamic manner in the whole topology and adapts to the dynamic change of the water environment [10].

Fingerprinting or scene analysis allows the estimation of a position by matching online measured data with pre-measured location-related data (i.e., power samples) [11,12]. Typically, two phases comprise a fingerprinting algorithm, i.e., (i) offline and (ii) online phase. The offline phase consists in the collection of location related data (e.g., the received signal strength) for each position in the environment. A location map is then built and stored. Then, in the online phase, the current measured data are matched to the measurements stored in the database in order to estimate a given position.

On the other hand, the needs of high data rates and low latency pushes towards Optical Wireless Communication (OWC) technologies and those on the visible range. Indeed, UVLC and, in particular, the range of green light ranging in [495–570] nm are of particular interest due to their low attenuation [13,14,15]. Typically, VLC technology has been scarcely applied for positioning-based applications in underwater scenarios. Indeed, Visible Light Positioning (VLP) systems have been largely applied in indoor scenarios [16,17], where it results in easier to install LED devices and noise can be strongly limited. VLP techniques use trilateration [18], angle-of-arrival [19,20] and time-of-arrival algorithms. Recently, Irshad et al. [21] presented a fingerprinting-based approach that exploits machine learning algorithms, working in both indoor and underwater scenarios. The proposed solution has been observed to achieve overall computational accuracy and an acceptable location estimation error, i.e., <1 m. Some important challenges have to be accounted for in the underwater VLP systems such as the noise due to wave motion, sunlight rays penetrating through water and existing obstacles such as fishes that need to be assimilated to moving obstacles. Another important factor to be accounted for is that the devices considered, i.e., LEDs and PhotoDiodes, are battery-powered and cannot rely on an existing infrastructure as in indoor scenarios. These factors need to be accounted for designing energy efficient localization algorithms. Another important challenging factor to be considered is that the localization is realized in a 3D scenario As a result, an efficient underwater VLP system should consider all these constraints [22]. In [23], a hybrid communication underwater system is developed that combines magnetic communication and VLC links. The former work independently of Line of Sight (LoS) requirements and turbidity but enables lower data rates than compared to VLC.

In this paper, we present extended simulation results of a footprinting-based localization algorithm for IoUT scenarios [24]. The proposed technique has been initially described in [24], where its effectiveness has been assessed by considering an ideal channel with no noise. The channel impulse response coefficients obtained from the LoS links of several LED transmitters are a priori collected in a database. The proposed UVLP system is comprised of a set of LED transmitters laying on the seabed, covering a given monitoring space by providing a given illumination level. A centralized architecture comprised of a RF device is exploited for localization computational tasks. Specifically, a RF sensor node is adopted for data storage and processing. The localization accuracy reaches the centimeter order (i.e., ≈20 cm) and variable errors due to the turbidity of the water. Independent from the localization algorithm, it is important to have a correct database of channel impulse responses that reflects the real underwater environment.

In this paper, we extend the proposed approach to consider a more realistic scenario, where the channel model includes a Gaussian noise distribution. In underwater optical wireless communication systems, there are several noise sources, including background noise, thermal noise, dark current noise and shot noise, which can be all modeled as the additive white Gaussian noise (AWGN) [25]. Specifically, we focus on the noise effect on the localization accuracy of the proposed algorithm expressed in terms of Mean Square Error. Simulation results have been carried out for different network configurations (i.e., by varying geometrical factors and the number of LEDs) and environmental scenarios (i.e., by varying the noise factor and the water type). As expected, the MSE suffers noisy environment and water turbidity, as well as on the cardinality of number of LEDs.

This paper is organized as follows. Section 2 presents the considered UVLP system model, while the proposed underwater localization algorithm is briefly described in Section 3. The effectiveness of this approach is addressed in Section 4 by using extensive simulation results expressed in terms of estimation position error. We observe the effect of noisy environment on the positioning error, which shows an increased trend for higher noise level. Moreover, as expected, best results are obtained when increasing the number of LEDs, as well as for higher beam angles. Finally, conclusions are drawn at the end of the paper.

## 2. System Model

**Figure 1:**
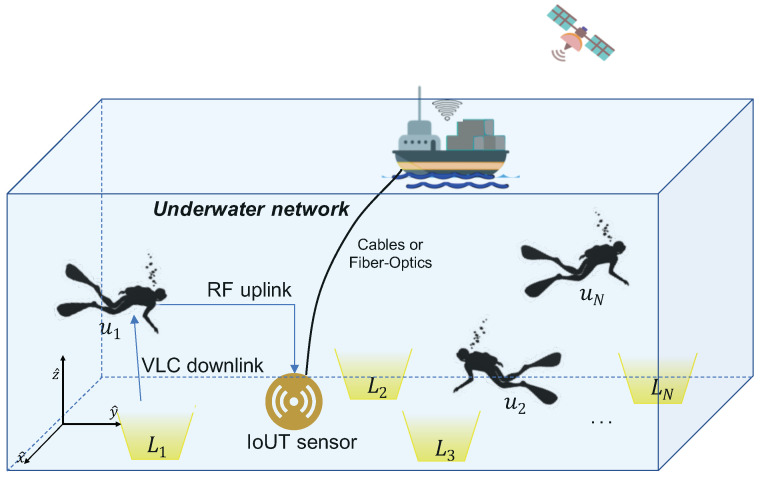
Schematic of a reference UVLC network, comprised of $L_{N}=4$ LED transmitters and a centralized RF IoUT sensor node. Wearable battery-powered photodetectors are assumed to be placed on the diver’s wetsuit.

Let us consider an UVLC network in which $L_{N}$ LED transmitters are installed in a given three-dimensional (3D) space S [m${}^{3}$], as exemplary shown in Figure 1 for $L_{N}=4$. Specifically, we consider $L=\{L_{1},L_{2},\ldots ,L_{N}\}$ to be the set of all LED transmitters that are assumed to be installed at the seabed, mainly in order to omit background noise from sunlight. Specifically, the *i*-th LED transmitter is posed on the seabed in a fixed known position, i.e., $P_{i}=\left(x_{i},y_{i},0\right)$ with $i=[1,2,\ldots ,L_{N}]$, where the *z*-axis refers to the vertical direction and the seabed is assumed to lay at z=0. This installation scheme, i.e., transmitters being located at the seabed, has several fundamental advantages over that when installing the transmitters closer to the sea surface. First, this scheme will reduce the expected noise and interference induced by sunlight, as sunlight is expected to penetrate through the sea surface only to a certain limit. Second, installing the transmitters at the seabed will also overcome the physical disturbance (instability) due to wave movement and other possible objects, e.g., boats, moving on the sea surface. Based on this last advantage, we lastly remark that the proposed installation scheme means that no further physical equipment will be needed to fix the transmitters in the planned locations.

As shown in Figure 1, we assume several users (e.g., divers) are moving in the monitoring space S, each of them equipped with a wearable photodetector (PD) placed on the wetsuit. Specifically, we consider $U=\{u_{1},u_{2},\ldots ,u_{M}\}$ with $u_{M}\neq 0$ as the set of PDs in the given area. We also assume that the PDs are battery-powered, lasting for a given time interval sufficient for diving tasks. Finally, the LED transmitters are able to illuminate the entire space S so that it is guaranteed a minimum illumination level at each position.

The network architecture of the UVLC scenario is shown in Figure 1, where a RF IoUT centralized sensor node is added due to its computational skills. Indeed, the RF IoUT node is accordingly deployed with the aim of performing simple computational tasks necessary for the localization algorithm. As it will be described in Section 3, a simple comparing operation is the only needed operation for localization computation, which then simplifies the hardware requirements of VLC devices. The IoUT sensor can collect and process the electrical signals at the output of the PDs in order to estimate the user position according to the proposed localization algorithm. The position estimation data are then transmitted to a control node (e.g., a boat in Figure 1) for monitoring and tracking tasks. Notice that the localization process is centralized; that is, the RF-based simple IoUT sensor is responsible for the entire localization process.

By assuming a LoS link between the *i*-th LED and the *j*-th PD, the output signal at the *j*-th PD is an electrical current Y(t) that can be expressed as follows:

(1) $$Y\left(t\right)=r\cdot X\left(t\right)*h_{w}\left(t\right)+N\left(t\right),$$

where X(t) is the transmitted optical intensity waveform of *T* time duration, *r* [A/W] is the responsivity of the *j*-th PD, $h_{w}\left(t\right)$ is the channel impulse response in the UVLC scenario and N(t) is the shot noise due to the random arrival of photons at the receiver. For sufficiently low rates, $h_{w}\left(t\right)$ can be approximated to the DC gain, i.e., $H_{w,0}$, which can be expressed according to Beer–Lambert law as follows [15]:

(2) $$\begin{array}{cc} H_{w,0}=e^{\left(-c\left(\lambda \right)d_{ij}\right)}\cdot & \\ \;\;\;\;\;\cdot \frac{A_{r}\left(m+1\right)}{2\pi d_{ij}^{2}}cos^{m}\left(\phi \right)T_{s}\left(\psi \right)g_{j}\left(\psi \right)cos\left(\psi \right)rect\left(\frac{\psi }{\psi_{C}}\right), & \end{array}$$

where $A_{r}$ (cm${}^{-2}$) is the active PD area, ϕ is the angle of irradiance, ψ is the angle of incidence w.r.t. the receiver axis and $T_{s}\left(\psi \right)$ and g(ψ) are the gains of the optical filter and of the optical non-imaging concentrator, respectively. We assume $T_{s}\left(\psi \right)=1$ and $g_{j}\left(\psi \right)=n^{2}/sin^{2}\left(\psi \right)$, with $\psi_{c}$ as the Field of View (FOV) of the *j*-th PD, i.e., the maximum angle at which the light emitted by the *i*-th LED is detected by the *j*-th PD and *n* as the refraction index. In addition, $m=-\ln\left(2\right)/\ln(cos\left(\psi_{1/2}\right))$ is the Lambertian order, where $\psi_{1/2}$ is the LED half intensity viewing angle and rect(x)=1 if $\left|x\right|\leq 1$ and rect(x)=0 otherwise.

Due to Beer–Lambert law, the influence of underwater environment on the LoS channel gains is provided by the the exponent c(λ) in Equation (2); in other words, we have the following:

(3) c(λ)=a(λ)+b(λ),

which represents the extinction coefficient, depending on the absorption coefficient, i.e., a(λ), and the scattering coefficient, i.e., b(λ), for a given wavelength λ (nm) and water type. From [26], different water types show specific extinction coefficients, such as pure sea, clear ocean, coastal water and harbor water, and they correspond, respectively, to [0.056,0.150,0.305,2.170] m${}^{-1}$, which are values of the extinction coefficient. Notice that higher (lower) values of the extinction coefficient correspond to turbid (non-turbid) water. Then, pure seas and clear oceans are considered as non-turbid water, whereas coastal and harbor water can be considered as turbid water [26]. It is expected that, due to water conditions affecting visibility, the position estimation error will be higher in turbid than in the case of non-turbid water.

## 3. Fingerprinting UVLP Algorithm

In this section, we describe our fingerprinting UVLP localization process as initially presented in [24]. This process is mainly composed of three major phases. Initially, the channel responses between each of the LEDs and each location within the entire space of interest are measured and stored in a map, which is also referred to as database. This database can be generated offline. Due to variable water conditions, it should be necessary to periodically update the database over certain time intervals.

It should be noted, that unlike the widely-investigated indoor environments in which the user moves within a two-dimensional (2D) space (normally defined as the (x,y) space in the Cartesian coordinates), a given user in the underwater environment can move within a 3D space, i.e., along the three *x*-, *y*- and *z*-coordinates. Thus, a 3D database would be generated and stored in the IoUT sensor. In order to facilitate building this database, we consider a spatial sampling concept in which the entire (3D) space is designed as a 3D grid and each point in this grid is considered as a possible user location. Specifically, the whole 3D space is modeled as $S=N_{x}\times N_{y}\times N_{z}$ samples, where the following is the case:

(4) $$N_{x}=l_{x}\cdot \Delta x,\;N_{x}=l_{x}\cdot \Delta x,\;\;\mathrm{and}\;N_{x}=l_{x}\cdot \Delta z,$$

where $l_{x,y,z}$ (m) are the space dimensions along {x,y,z} directions and Δ{x,y,z} (with 0<Δ{x,y,z}≤1) are the sampling rates along the corresponding orthogonal directions. Notice that the lower Δ{x,y,z} is, the higher the accuracy of the localization algorithm will be. Indeed, reducing the gap between adjacent points in this grid will result in a higher (smaller) localization accuracy (error). However, this would require larger memory to store this map, which can be observed as a hardware limitation as simple IoUT devices are expected to have a very limited memory.

Having the database stored at the IoUT device, a localization algorithm can be implemented in the second phase by using that database. We consider a simple but still efficient localization algorithm in which we measure the channel responses between the four LEDs and the user (unknown) location to be estimated and compare these values with those stored at the database. We then use the clear mapping between channel gains and locations in the database to estimate the user location. The main challenge of the proposed localization technique is to reduce the number of locations that can be considered as potential user locations. In order to handle this issue, we consider a recursive algorithm in which we aim at minimizing the set of possible user locations at each localization algorithm step.

Before proceeding, we should stress that the recursive algorithm detailed below is based on the assumption that the channel gain contributions of each LED at the (unknown) user location are estimated beforehand. This assumption is indeed required by other algorithms based on the received signal strength indicator (RSSI). However, the localization algorithm described here, which is based on a stored database, has a fundamental advantage that no requirements are needed on the number of LEDs covering the user location to guarantee good localization accuracy. For instance, the triangulation algorithm based on the RSSI requires a minimum of three APs to obtain reasonable results. Thus, the algorithm in this paper is expected to obtain reasonable results with much less LEDs covering the entire space.

Having this important remark in mind, we now describe the proposed localization algorithm. Let us assume four LED transmitters are deployed in the underwater scenario as depicted in Figure 1. The *i*-th user (i.e., $u_{i}$) is being located at an unknown location $P_{i}=\left(x_{i},y_{i},z_{i}\right)$, then the target is to obtain the location estimate $\hat{P}=\left({\hat{x}}_{i},{\hat{y}}_{i},{\hat{z}}_{i}\right)$. To this end, the proposed localization algorithm goes through the following three steps, i.e., (i) database construction, (ii) likely position detection and (iii) position estimation.

### 3.1. Step I. Database Construction

This first step corresponds to the construction of an underwater scenario channel gain database, namely D, which is then stored in the RF sensor node. Let D carry all the channel responses between all LED transmitters and all the possible user locations within the underwater environment as defined in 3D space S; in other words, we have the following:

(5) $$D=\left[h_{1}^{\left(S\right)},h_{2}^{\left(S\right)},\ldots ,h_{L_{N}}^{\left(S\right)}\right],$$

where $L_{N}$ is the number of APs and the *j*-th h with $j=1,2,\ldots ,L_{N}$ is a 3D matrix of dimensions $N_{x}\times N_{y}\times N_{z}$ for which its elements are the channel impulse responses from the *j*-th LED. The database D is updated in a timely manner in order to compute the position estimation as correctly relative to the real scenario. Indeed, if the database collects channel impulse responses from a water environment type that does not reflect the real scenario, then the position estimation can be badly affected. Finally, the database is stored in the RF sensor local cache.

### 3.2. Step II. Likely Position Detection

For a given unknown position (i.e., $P_{i}$) of the *i*-th user within the monitoring space S, in this step the algorithm aims at identifying a subset of possible user locations based on the measured channel gains between $P_{i}$ and all APs. In addition to the possible localization error when estimating the user position, there is another source of error that should not be ignored, which is the channel gain estimation error. As already mentioned, the localization algorithm in this paper is based on the assumption that the channel gains between all APs and $P_{i}$ are known in advance.

However, in practice we cannot have perfect measures for the channel gains and only channel estimates are possible. This is indeed due to the different noise sources affecting the received signals. In order to mathematically regard the impact of imperfect knowledge on the channel gains, we consider the following model for the estimated channel gain between $P_{i}$ and the *j*-th AP, i.e., ${\tilde{h}}_{j}^{P_{i}}$:

(6) $${\tilde{h}}_{j}^{P_{i}}=h_{j}^{P_{i}}+{\hat{h}}_{j}^{P_{i}},$$

where $h_{j}^{P_{i}}$ is the true channel gain as expressed in Equation (2) and ${\hat{h}}_{j}^{P_{i}}$ is a term that accounts for the channel estimation error. For the sake of simplicity and without any loss of generality, in this paper we assume that the estimation error ${\hat{h}}_{j}^{P_{i}}$ follows a zero-mean Gaussian distribution with a finite and known variance $\sigma_{\hat{h}}^{2}$.

Now, let us denote the vector carrying the estimates of the channel impulse responses between all LEDs and the position $P_{i}$ as ${\tilde{h}}^{\left(P_{i}\right)}$; in other words, we have the following.

(7) $${\tilde{h}}^{\left(P_{i}\right)}=\left[{\tilde{h}}_{1}^{\left(P_{i}\right)},{\tilde{h}}_{2}^{\left(P_{i}\right)},\cdots ,{\tilde{h}}_{L_{N}}^{\left(P_{i}\right)}\right].$$

Notice that the smaller the distance between $P_{i}$ and a given LED, the larger the corresponding channel gain.

The position estimation algorithm starts by comparing the channel gains at the database to the ones estimated along the user path. As a result, the algorithm will form several subsets of the database, collecting the possible user locations until reaching the position estimation. Let us assume $D^{\left(1\right)}\subset D$ as the first subset of the possible user locations, obtained by comparing the channel gains at the database, i.e., $h_{\ell }^{\left(D\right)}$ for $\ell =\{1,2,\ldots ,L_{N}\}$, with the largest measured channel gain, i.e., ${\tilde{h}}_{l}^{\left(P_{i}\right)}=\max{\tilde{h}}^{\left(P_{i}\right)}$. Specifically, the subset $D^{\left(1\right)}$ contains all the user locations whose channel responses satisfy the following criteria:

(8) $$-\xi_{h}\leq h_{\ell }^{\left(D\right)}-{\tilde{h}}_{l}^{\left(P_{i}\right)}\leq \xi_{h},$$

where $\xi_{h}$ is a predefined deviation factor.

The subset $D^{\left(1\right)}$ will be further updated in order to reduce the number of possible user locations. In particular, the channel gains at the subset $D^{\left(1\right)}$ will be compared with the second largest channel gain in ${\tilde{h}}^{\left(P_{i}\right)}$. As a result, the second subset $D^{\left(2\right)}\subset D^{\left(1\right)}$ will be created, which contains all the user locations whose channel responses match the following limits:

(9) $$-2\xi_{h}\leq h_{\ell }^{\left(D^{\left(1\right)}\right)}-{\bar{h}}_{l}^{\left(P_{i}\right)}\leq 2\xi_{h},$$

where ${\bar{h}}_{l}^{\left(P_{i}\right)}$ is the updated (second) largest channel gain in ${\tilde{h}}^{\left(P_{i}\right)}$. In order to further reduce the set of possible user locations, subset $D^{\left(3\right)}\subset D^{\left(2\right)}$ is created with all the locations of the *i*-th user whose channel gains satisfy Equation (9) with ${\bar{h}}_{l}^{\left(P_{i}\right)}$ no being the third largest channel gain in ${\tilde{h}}^{\left(P_{i}\right)}$ and $h_{l}^{\left(D^{\left(1\right)}\right)}$ being replaced with $h_{l}^{\left(D^{\left(2\right)}\right)}$. This step will keep running until the smallest element in ${\tilde{h}}^{\left(P_{i}\right)}$, which will result in the subset $D^{\left(L_{N}\right)}\subset D^{\left(L_{N}-1\right)}$, where $L_{N}$ is the number of LEDs.

**Remark** **1.** *In practical systems, notice that the estimation error mainly depends on the algorithm used for the channel estimation process and it is expected to be dependent on many parameters, in particular, on all parameters defining the received signal-to-noise-and-interference ratio (SINR). As the channel estimation phase is beyond the scope of this paper and in order to provide general analyses that only focus on the localization phase, we then made the assumption of modeling the estimation error as a random variable with predefined and known parameters. However, for the sake of completeness, let us briefly show how the estimation error can be formulated by considering a certain estimation algorithm. From Equation (2), we notice that the distance between the transmitter and receiver is the only unknown parameter that should be estimated. Subsequently, the process of channel estimation is equivalent to estimating the corresponding distance. This implies that the estimation error depends on the used distance estimation problem. To this end, we consider the task of estimating the position of the k-th user (location), which is covered by the AP(s) for which their index(es) is(are) defined by the set B. Furthermore, we can consider an estimation algorithm based on the received signal strength (RSS) due to its simplicity. As a performance measure, we adopt the Cramer-Rao bound (CRB), which defines the lower bound on the variance of any unbiased estimator [27].*

Mathematically speaking, let $P_{j}$ be the transmission power of the *j*-th AP and $h_{j,k}$ be the channel gain between the *k*-th user and the *j*-th AP. By using the CRB, the estimator error related to the distance $d_{j,k}$ has the following lower bound [28]:

(10) $$E=\sum_{j\in B}{\left(-\frac{\partial h_{j,k}}{\partial d_{j,k}}\right)}^{-1}\frac{\sigma_{j,k}}{\alpha P_{j}},$$

where $\frac{\partial h_{j,k}}{\partial d_{j,k}}$ is the partial derivative of the channel gain $h_{j,k}$ with respect to the distance $d_{j,k}$, $\sigma_{j,k}^{2}$ is the additive noise variance and α is the optical-to-electrical conversion efficiency. Notice that Equation (10) follows from the fact that the error of estimating the distance to a given AP is independent of the error caused when estimating the distances to the other APs.

**Remark** **2.** *From the above analysis, notice that we considered the impact of channel estimation error when running the algorithm but not when constructing the database. This is basically due to the fact that the conditions when constructing the database have two fundamental differences from the others when running the algorithm. First, constructing the database is performed offline and, thus, it does not have the time constraints as those imposed when running the algorithm, which should be executed in real-time. Second, the user locations are perfectly known when constructing the database. With these two aspects, it is safe to assume a near-perfect channel estimation when building the database. For example, having no time constraints implies that multiple measurements can be observed for the same position and so the noise impact can be eliminated.*

### 3.3. Step III. Position estimation

Lastly in this step, the algorithm estimates the user position using the final subset of the possible locations $D^{\left(L_{N}\right)}$. To this end, the algorithm calculates several estimates ${\hat{P}}_{j}$, where $j=1,2,\ldots ,|D^{\left(L_{N}\right)}|$ and $|D^{\left(4\right)}|$ refers to the cardinality of the subset $D^{\left(L_{N}\right)}$, as follows:

(11) $${\hat{P}}_{j}=\sum_{l=1}^{L_{N}}\left|{\tilde{h}}_{l}^{\left(P_{i}\right)}-h_{\ell ,j}^{\left(D^{\left(L_{N}\right)}\right)}\right|,$$

where ${\tilde{h}}_{l}^{\left(P_{i}\right)}$ is the measured channel gain between the *l*-th LED and the *i*-th user location, as defined in Equation (7), and $h_{\ell ,j}^{\left(D^{\left(L_{N}\right)}\right)}$ is the stored channel gain between the *ℓ*-th LED and the *j*-th user location at the subset $D^{\left(L_{N}\right)}$. In Equation (11), the estimations are represented as the sums of differences among the channel gains at the receiver and those in the subset $D^{\left(L_{N}\right)}$. The algorithm then selects the user locations with the lowest ${\hat{P}}_{j}$ values (i.e., $P_{Rx_{m_{1}}},P_{Rx_{m_{2}}}$) and the estimated user location will be the corresponding average; in other words, the following is the case:

(12) $$\begin{array}{cc} {\hat{P}}_{i} & =\mathrm{avg}\{P_{Rx_{m_{1}}},P_{Rx_{m_{2}}}\}\\ \; & =\left[\frac{x_{Rx_{m_{1}}}+x_{Rx_{m_{2}}}}{2},\frac{y_{Rx_{m_{1}}}+y_{Rx_{m_{2}}}}{2},\frac{z_{Rx_{m_{1}}}+z_{Rx_{m_{2}}}}{2}\right], \end{array}$$

where $P_{Rx_{m_{1}}}=\left[x_{Rx_{m_{1}}},y_{Rx_{m_{1}}},z_{Rx_{m_{1}}}\right]$ and $P_{Rx_{m_{2}}}=\left[x_{Rx_{m_{2}}},y_{Rx_{m_{2}}},z_{Rx_{m_{2}}}\right]$. Notice that the indexes $m_{1}$ and $m_{2}$ refer to those as defined in the stored database. In the special case when the subset $D^{\left(L_{N}\right)}$ has only a single possible position, i.e., $D^{\left(L_{N}\right)}=\left\{P_{Rx_{k}}\right\}$, then the algorithm simply selects $P_{Rx_{k}}$ as the estimated user location; in other words, the following is the case.

(13) $${\hat{P}}_{i}=P_{Rx_{k}}.$$

Again, notice that the position index *k* refers to the *k*-th user position as defined in the (original) stored database, as the corresponding index with respect to the subset $D^{\left(L_{N}\right)}$ will always be one for this specific case.

The effectiveness of the localization algorithm is then computed through the estimation error of the *i*-th user position $P_{i}$, for different *x*, *y* and *z* directions, i.e., $E_{\left\{x,y,z\right\}}^{\left(P_{i}\right)}$ (m), defined, respectively, as follows.

(14) $$E_{x}^{\left(P_{i}\right)}={\hat{x}}_{i}-x_{i},\;E_{y}^{\left(P_{i}\right)}={\hat{y}}_{i}-y_{i},\;E_{z}^{\left(P_{i}\right)}={\hat{z}}_{i}-z_{i}.$$

Based on specific applications, the accuracy of the localization algorithm is reflected in the following constraint:

(15) $$E_{\left\{x,y,z\right\}}^{\left(P_{i}\right)}\leq \zeta ,$$

where ζ is the localization upper threshold. As an instance, in case of environmental monitoring, we can assume ζ=20 cm.

Finally, from Equation (14), we can derive the MSN factor (m) of the *i*-th user position $P_{i}$, computed as follows.

(16) $$MSE^{\left(P_{i}\right)}=\sqrt{{E_{x}^{\left(P_{i}\right)}}^{2}+{E_{y}^{\left(P_{i}\right)}}^{2}+{E_{z}^{\left(P_{i}\right)}}^{2}}.$$

## 4. Results

In this section, we present the extended simulated results by assessing the proposed localization algorithm in the case of Additive White Gaussian Noise (AWGN) distribution with zero mean value and standard deviation $\sigma_{n}$, i.e., $N(0,\sigma_{n})$, in the monitoring space. Specifically, we show how the noise can affect the positioning estimation, expressed in terms of Mean Square Error (MSE). Different scenarios have been evaluated in the case of varying (i) the number of LEDs deployed in the given environment, (ii) the additive noise and (iii) the water types. We can observe that—as expected—the MSE is strongly affected by the number of LED transmitters covering the monitoring space, as well as by the AWGN. It is, thus, necessary to mitigate the noise by means of noise cancellation techniques. The noise mitigation techniques are of paramount importance in VLC systems and, above all, for localization techniques, where noise could negatively impact on the accuracy of localization. In [29,30], the authors have demonstrated a technique for mitigating the impact of noise by listening to the environment for a certain time and inferring the specific features of the noise. Based on the statistical characteristics of the noise, by applying Yule–Walker equations, it has been possible to drastically reduce the impact of the noise.

**Figure 2:**
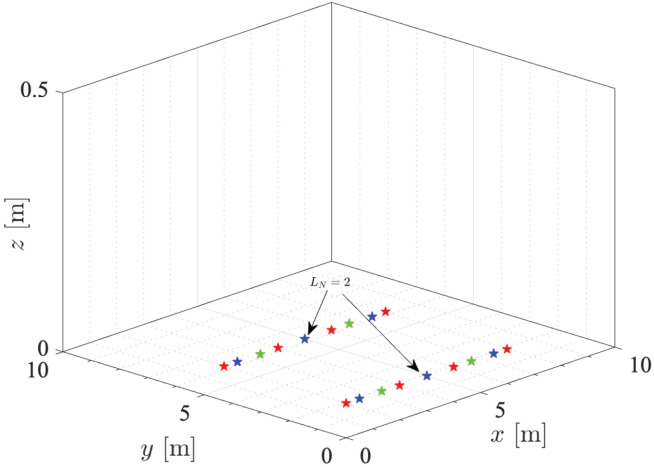
Map of different LED transmitter scenarios deployed in the seabed following a grid structure. Green, blue and red stars refer to $L_{N}=\left[4,6,8\right]$, respectively. The case of $L_{N}=2$ overlaps with two LEDs of the $\left(L_{N}=6\right)$ LED configuration.

Figure 2 depicts the different network configurations used in the simulation results for variable number of LEDs, i.e., $L_{N}=\left[2,4,6,8\right]$. Notice that LEDs are randomly deployed in the whole space according to a grid configuration. Specifically, the *i*-th LED is positioned in $\left(x_{i},y_{i},0\right)$ assuming an inter-LED distance (m); it is expressed as follows:

(17) $$d_{\left(i,i+1\right)}=l_{x}/\left(L_{N}/2+1\right).$$

All the LEDs transmit the same power level $P_{t}=10$ Watt. All the parameters used to generate the simulation results are collected in Table 1.

**Figure 3:**
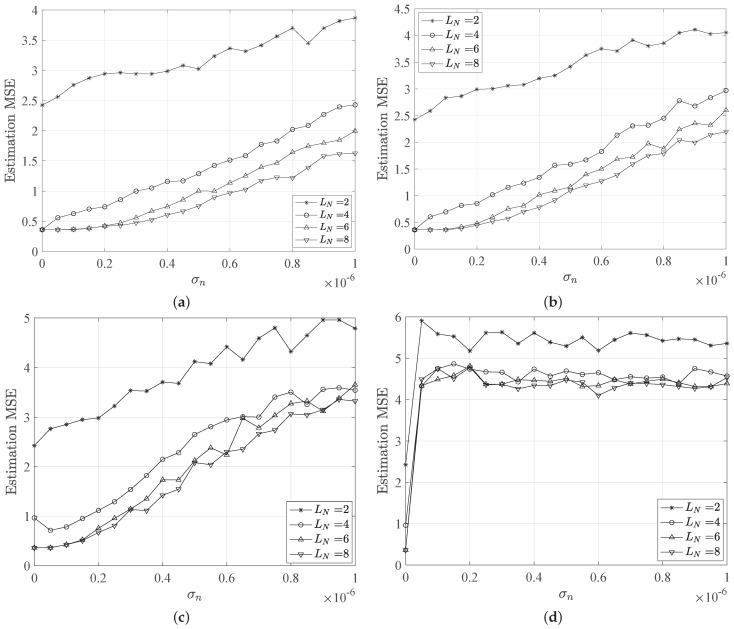
MSE of the position estimation behavior versus the noise standard deviation for different network configurations and water types, i.e., (**a**) pure, (**b**) clear, (**c**) coastal and (**d**) harbor water. We assume a beam angle of $50^{\circ }$.

**Figure 4:**
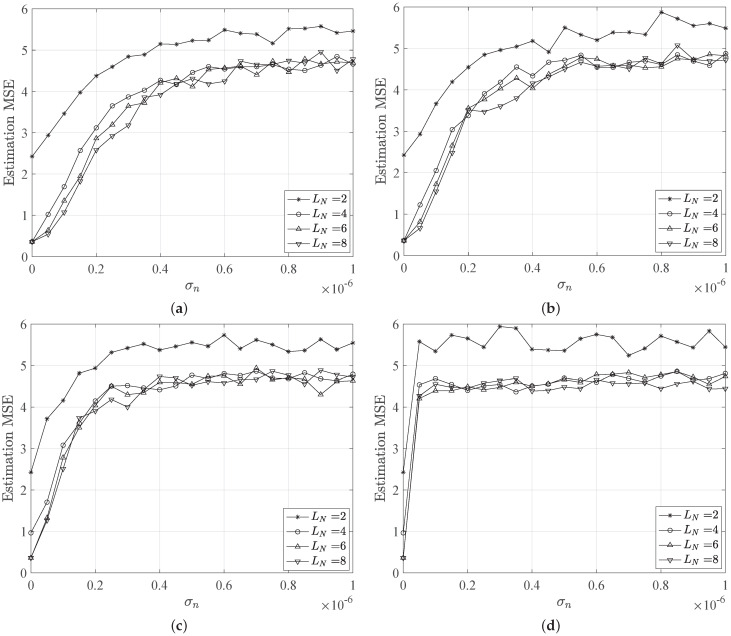
MSE of the position estimation behavior versus the noise standard deviation for different network configurations and water types, i.e., (**a**) pure, (**b**) clear, (**c**) coastal and (**d**) harbor water. We assume a beam angle of $80^{\circ }$.

Figure 3 and Figure 4 show the MSE trends versus the noise standard deviation $\sigma_{n}$ for different LED configurations and beam angles. As expected, the MSE shows an increasing behavior for increasing noise, as well as a reduction due to higher number of LEDs deployed. Specifically, we observe high MSE values for the case of only $L_{N}=2$ LEDs, which represents the worst scenario for localization purpose. Indeed, in this LED configuration, the estimation error occurs as a result of the error in estimating the distances, which are the distances to both LEDs, as well as the localization ambiguity error. Thus, also for $\sigma_{n}=0$, the configuration $L_{N}=2$ provides a MSE equal to ≈2.5 m, which is not acceptable for common localization applications requiring centimeter accuracy.

Interesting, the MSE keeps an almost flat behavior for small values of $\sigma_{n}$ for $L_{N}=\left[6,8\right]$ scenarios. Here, the MSE value is ≈0.5 m, which is the most acceptable. This is noticeable for turbidity values corresponding to pure, clear and coastal waters, while for harbor water the MSE shows a very fast increasing behavior for small values of $\sigma_{n}$ and keeps high values for increasing noise. In this case, shown in Figure 3d, the water turbidity strongly affects the localization estimation, providing MSE values higher than 4 m.

Similar considerations applied to the case of increased LED beam angles, i.e., $\theta =80^{\circ }$, except that it induces a degradation of the MSE performance. Indeed, higher beam angles can provide a less accurate localization estimation, since the LED coverage areas will be larger. In the case of no noise, i.e., $\sigma_{n}=0$, the MSE is still comparable to the previous case of $\theta =50^{\circ }$, but small variations of noise standard deviation correspond to fast increasing MSE values. In the worst case corresponding to $\sigma_{n}=10^{-6}$, the MSE reaches ≈5 (m) for all LED configurations and independently from the considered water type. On the other side, in Figure 3, the MSE reaches lower values that are easily distinguishable for different water types.

**Figure 5:**
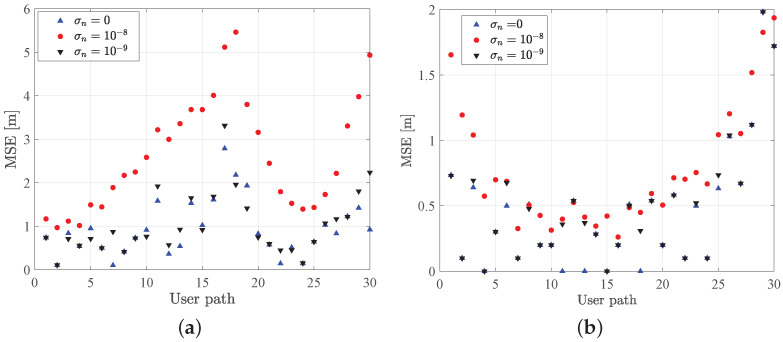
Comparison of MSE trends for different values of noise standard deviation computed for different user positions, for (**a**) variable and (**b**) fixed position along *z*-direction, i.e., z=4 m.

**Figure 6:**
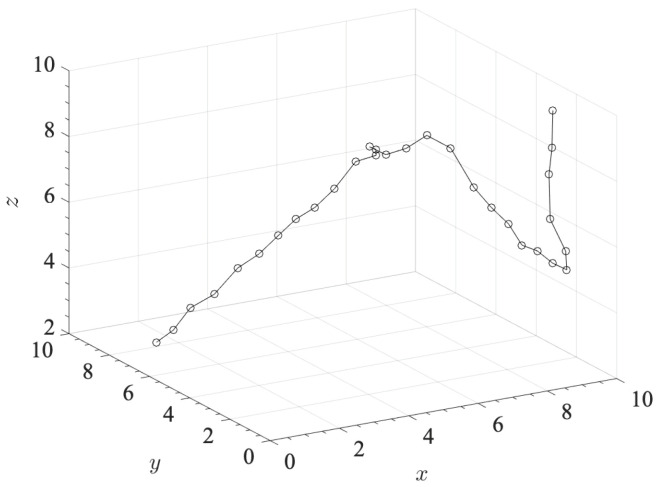
User path within the monitoring space S.

In Figure 5, we compare the MSE values sampled along a given user’s path, as depicted in Figure 6. MSE trends are obtained assuming different values of noise standard deviation, i.e., $\sigma_{n}=\left[0,10^{-8},10^{-9}\right]$, in case of $L_{N}=4$ and pure water. Furthermore, we distinguish two cases, i.e., assuming (i) a variable and (ii) a fixed user position along *z* direction. In Figure 5a, we observed higher values of MSE for increasing noise, while in the absence of noise the MSE is kept lower than 1 m along the user path. On the other side, in Figure 5b, we observed that assuming the user does not move along the *z*-direction, i.e., $z_{i}=4$ m, strongly reduces the MSE component along the *z*-direction and also the overall MSE value.

**Figure 7:**
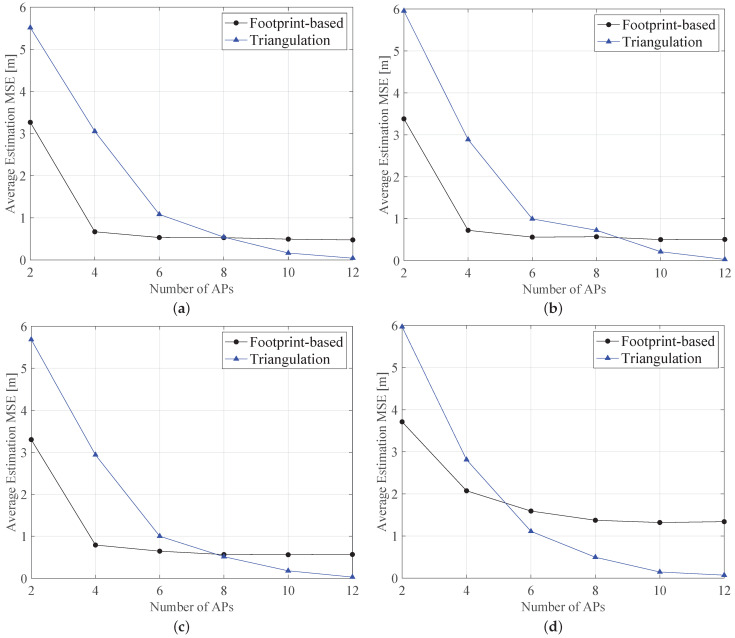
Average MSE comparison of fingerprint to triangulation approaches versus the number of APs and for different water types, i.e., (**a**) pure, (**b**) clear, (**c**) coastal and (**d**) harbor water. We assume a beam angle of $60^{\circ }$.

Furthermore, for comparison purposes, in Figure 7 we demonstrated the performance of the proposed fingerprinting-based approach as compared to the well-known triangulation: In the case of a variable number of APs (i.e., from 2 to 12) randomly deployed in the considered scenario. As expected for both two cases, in increasing number of APs, the estimation error decreases, since the environment is covered by a higher number of devices. Moreover, in increasing the value of the extinction coefficient, the error is higher than compared to the case of low-turbidity water. The comparison of triangulation to the fingerprinting approach shows a variable behavior, where, initially, the lower number of APs in the proposed approach outperforms the triangulation technique. In increasing the number of APs, the triangulation approach shows better performance than the fingerprinting one. This occurs at ≈8 APs, while this threshold lowers at ≈6 APs for harbor water (see Figure 7c). Furthermore, the variation of the average MSE obtained with the fingerprinting approach is shorter than the triangulation case. For instance, in the case of harbor water in Figure 7d, the average MSE obtained with the fingerprinting solution ranges from ≈3.71 m to ≈1.34 m, while the triangulation method showed higher range (i.e., from ≈6 m for $L_{N}=2$ to ≈0.1 m for $L_{N}=12$).

Finally, in this section we illustrate the impact of noise on the distance estimation error. We particularly highlight the impact of the solar noise, i.e., due to the sunlight, on the estimation performance. As shown in Equation (10), the system noise affects the estimation error lower bound through the noise variance $\sigma_{j,k}^{2}$. Considering the solar noise, we can then express $\sigma_{j,k}^{2}$ as follows [31]:

(18) $$\sigma_{j,k}^{2}=\frac{\pi^{2}D^{2}\Psi_{c}^{2}\Delta \lambda L_{s}}{4},$$

where *D* (m) is the diameter of the photodiode aperture, Δλ is the optical filter bandwidth and $L_{s}$ is the up-welling solar radiance, which is provided by the following:

(19) $$L_{s}=\frac{ERL_{fac}e^{-c\cdot d_{w}}}{\pi },$$

with *E* as the down-welling irradiance, *R* as the underwater reflectance, $L_{fac}$ as the factor describing the directional dependence of the underwater radiance and $d_{w}$ (m) is the water depth, i.e., the distance between the water surface and the receiver. In this case, if the total water depth is $d_{w,tot}$ (m), then $d_{w}=d_{w,tot}-r_{v}$, where $r_{v}$ (m) is the vertical distance between the transmitting and receiving planes.

**Figure 8:**
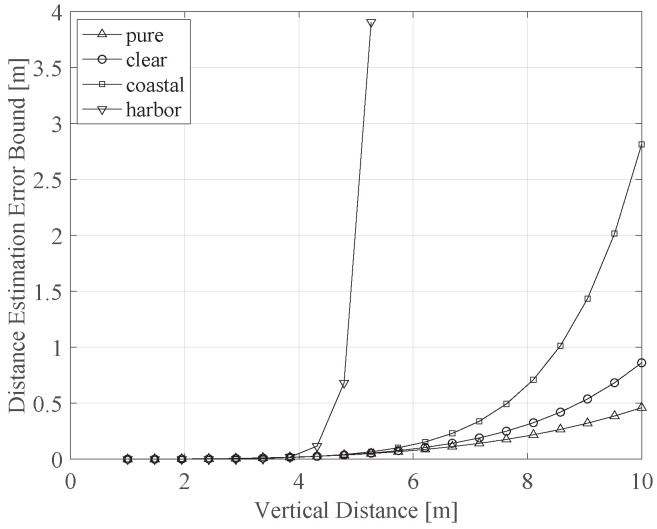
Maximum achievable estimation error (m) versus the vertical distance, for different water types.

The effect of sunlight noise on the estimation algorithm is expressed in Figure 8, which describes this effect as a function of the vertical distance $r_{v}$ and for different water conditions, assuming E=1440 (W/m${}^{2}$), R=1.25 and Δλ=20 (nm). We also assume a total water depth of 10 m. As clearly observed, increasing the vertical distance results in a drastic increase in the estimation error, especially as the distance goes beyond a certain value, e.g., when the distance is greater than ≈6 m for coastal water and ≈4 m for harbor water. This is, indeed, expected, as increasing $r_{v}$ means that the user is getting closer to the water surface and, hence, the sunlight is getting brighter. Notice that a vertical distance of 10 m means that the user is located directly on the water surface, while the transmitters are located on the seabed.

## 5. Conclusions

This paper has investigated the noise effect on a fingerprinting-based localization VLC approach for underwater scenarios. The proposed approach has been initially presented in [24] and, here, novel simulation results have been extended, dealing with noise effect and different network configurations. We have observed how the noise distribution can strongly affect the localization estimation, as well as the LED deployment and water turbidity. As a conclusion, we can state that an efficient localization approach (i.e., providing centimeter-order accuracy) needs to consider an optimized LED deployment, as well as noise cancellation techniques. In particular, the characterization of the statistical characteristics of the noise can be exploited to apply efficient mitigation techniques.

## Figures and Tables

**Table 1:** Main parameters used in the simulations.

Parameter	Values
LED Power	10 W
Beam angle	${\left[50,80\right]}^{\circ }$
Monitoring space S $\left(l_{x}\times l_{y}\times l_{z}\right)$	10 m × 10 m × 10 m
PD field of view (FOV), $\psi_{C}$	$60^{\circ }$
Refractive index, *n*	1.5
Optical filter gain, $T_{s}\left(\psi \right)$	1
Effective PD area, $A_{e}$	1 cm${}^{2}$
Noise standard deviation $\sigma_{n}$	$\left[0,10^{-6}\right]$
Number of LEDs	[2,4,6,8]
Extinction coefficient, *c*	[0.056,0.150,0.305,2.170] m${}^{-1}$
